# Exercise Stress Testing Enhances Plasma Protein Carbonyl Levels in Patients With Asymptomatic Moderate-to-Severe Aortic Stenosis

**DOI:** 10.1155/omcl/4852300

**Published:** 2024-12-20

**Authors:** Magdalena Kopytek, Renata Kolasa-Trela, Krzysztof Piotr Malinowski, Michał Ząbczyk, Joanna Natorska, Anetta Undas

**Affiliations:** ^1^Department of Thromboembolic Disorders, Institute of Cardiology, Jagiellonian University Medical College, 80 Pradnicka St. 31–202, Krakow, Poland; ^2^Krakow Centre for Medical Research and Technologies, St. John Paul II Hospital, 80 Pradnicka St. 31–202, Krakow, Poland; ^3^Department of Diagnostic Medicine, St. John Paul II Hospital, 80 Pradnicka St. 31–202, Krakow, Poland; ^4^Department of Bioinformatics and Telemedicine, Faculty of Medicine, Jagiellonian University Medical College, 7 Medyczna St. 30–688, Krakow, Poland; ^5^Center for Digital Medicine and Robotics, Jagiellonian University Medical College, 7E Kopernika St. 31–034, Krakow, Poland

**Keywords:** aortic stenosis, clot lysis time, exercise stress test, interleukins, oxidative stress, protein carbonyl

## Abstract

**Background:** Exercise stress test-induced hypofibrinolysis and changes in circulating levels of several interleukins have been observed in aortic stenosis (AS). However, it is unknown whether the pattern of exercise-induced changes in oxidative stress differs between AS patients and controls and if the differences are associated with changes in fibrinolysis and inflammation.

**Methods:** We studied 32 asymptomatic patients with moderate-to-severe AS and 32 controls of similar age, sex, and body mass index. We assessed plasma protein carbonyl (PC) concentrations, a marker of oxidative stress, in relation to interleukin (IL)-10 and -6 levels and fibrinolysis capacity, expressed as plasma clot lysis time (CLT) at four time points: at baseline, at peak exercise, 1 and 24 h after a symptom-limited exercise test.

**Results:** AS patients had 12.8% and 27% higher PC concentrations 1 and 24 h after exercise than controls (both *p*  < 0.05), with no differences at baseline and peak exercise. In AS patients, PC concentration was 8.3% higher at peak exercise compared to baseline followed by further PC increase (+12.8% at 1 h and +20.5% at 24 h) compared to peak exercise (all *p*  < 0.05). In controls, PC concentrations increased during exercise, reaching the highest values 1 h after exercise (+21.9%). In the AS group, PC concentrations at baseline correlated with AS severity measured as peak transvalvular velocity (*V*_max_: *r* = 0.49, *p*  < 0.05), mean (PG_mean_: *r* = 0.42, *p*  < 0.05), and maximal transvalvular pressure gradients (PG_max_: *r* = 0.41, *p*  < 0.05). PC concentrations correlated with IL-10 levels 1 h (*r* = 0.37, *p*  < 0.05) and 24 h (*r* = 0.38, *p*  < 0.05) post exercise in AS patients, whereas in controls only at baseline (*r* = 0.42, *p*  < 0.05). No associations between PC levels and IL-6 or CLT were observed at any time point.

**Conclusions:** Our findings show that AS patients respond differently to exercise in terms of PC compared to controls, which suggests a novel effect of hemodynamic abnormalities in this disease on intensity of oxidative stress.

## 1. Introduction

Aortic stenosis (AS) is a common valvular heart disease with no effective pharmacological therapy that reverses or delays disease progression [1]. The pathophysiology of AS is complex and tightly regulated, involving multiple pathways that are only partially understood [2]. Data indicated that enhanced oxidative stress may accelerate the development of AS through lipoproteins infiltration, inflammation, and fibro-calcification, which are pivotal processes implicated in the pathophysiology of AS [3]. Protein carbonylation is an irreversible post-translational modification of amino acids at the side chains of lysine, arginine, proline, or threonine residues induced by reactive oxygen species (ROS) [4]. Protein carbonyl (PC) is considered as a reliable biomarker of oxidative stress associated with cellular damage, aging, and diverse age-related disorders [5, 6]. Siudut et al. [7] have demonstrated in patients with severe AS that PC levels are associated with the disease severity measured by echocardiography. Furthermore, PC levels positively correlated with clot lysis time (CLT), plasminogen activator inhibitor-1 (PAI-1), and tissue factor (TF) concentrations, suggesting that increased oxidative stress contributes to impaired fibrinolysis and enhanced coagulation activation in AS, which may be associated with disease progression [7]. Since it is known that exercise is associated with a reduced risk to develop cardiovascular disease, regular physical activity is strongly advocated for individuals with existing cardiovascular disease and those with cardiovascular risk factors related to lifestyle [8]. Kolasa-Trela et al. [9] have reported that exercise stress testing induced prothrombotic alterations evidenced by enhanced endogenous thrombin potential and impaired fibrinolytic capacity in asymptomatic moderate-to-severe AS patients. In addition, exercise testing in these patients induced also changes in interleukins (IL-6, IL-10) and growth factors levels [10]. Interestingly, most studies, but not all [11, 12], have reported increased plasma PC concentrations following exercise compared to baseline values [13–16], which was explained by the fact that exercise may stimulate the clearance of PC in plasma present at baseline with simultaneous increasing ROS production that promotes the formation of new PC groups [17]. To our knowledge, it is unknown how AS affects PC during exercise test. We aimed to investigate whether the profile of exercise-induced changes in plasma PC concentrations differ between patients with asymptomatic AS free of significant atherosclerotic vascular disease and matched control subjects and if such changes are associated with fibrinolysis and inflammation markers in circulating blood.

## 2. Materials and Methods

### 2.1. Patients

In this case–control study, 32 consecutive asymptomatic patients with AS were compared with 32 control individuals from the families of hospital personnel of similar age, sex, and BMI. All participants were Caucasian, and they were recruited from March 2011 to June 2012 at the St. John Paul II Hospital, Krakow, Poland. The study groups were described in detail previously [9, 10]. Briefly, asymptomatic patients with moderate-to-severe AS were recruited in accordance with the ESC/EACTS guidelines for the management of valvular heart disease [18]. However, the severity of AS was defined based on indexed AVA (AVAI), with moderate AS classified as AVAI > 0.6 and ≤ 0.85 cm^2^/m^2^ and severe AS as AVAI ≤ 0.6 cm^2^/m^2^ [19]. The main exclusion criteria were myocardial infarction, stroke, atrial fibrillation or venous thromboembolism, dizziness, or syncope, another cardiac valve disease of more than a moderate degree, left ventricular ejection fraction (LVEF) <50%, diabetes mellitus treated with insulin, severe renal or liver dysfunction, the use of nonsteroidal anti-inflammatory drugs other than aspirin, and inability to perform exercise testing. Definitions of comorbidities were shown previously [9, 10]. To evaluate the extent of atherosclerotic vascular disease, intima-media thickness in both right and left common carotid artery was measured in accordance with the Mannheim carotid intima-media thickness consensus [20].

The Ethics Committee (Krakow District Medical Chamber, Poland) approved the study, and all participants provided written informed consent in accordance with the Declaration of Helsinki.

### 2.2. Exercise Test

Symptom-limited exercise stress echocardiography was performed on a bicycle ergometer (Ergoline, Bitz, Germany) in a semi supine position with a continuous examination by one experienced cardiologist. Transthoracic echocardiography was performed in both AS and control participants using the Philips iE33 (Philips Electronics, Andover, MA, USA) ultrasound apparatus, and the following parameters were evaluated: LVEF, AVA, peak transvalvular velocity (*V*_max_), mean, and maximal transvalvular pressure gradients (PG_mean_, PG_max_). After 3 min of the initial workload of 25 W, the workload was increased every 3 min by 25 W [21]. Electrocardiogram was monitored, and blood pressure was measured every 3 min during exercise. Exercise was stopped in case of typical chest pain, dyspnea, dizziness, fatigue, hypotension, ventricular arrhythmia, when the age-related maximum heart rate was reached, or on the patient's demand. Echocardiography was performed at rest and peak exercise.

### 2.3. Laboratory Analysis

Fasting venous blood was drawn from the antecubital vein in AS patients and control participants. Citrated blood (9:1 of 0.106 M sodium citrate) was centrifuged at 2500*g* for 20 min at 20°C, while blood drawn into EDTA or serum tubes was centrifuged at 1600*g* for 10 min at 4°C. All blood samples were stored at −80°C until analysis. Routine laboratory assays were used to determine glucose, creatinine, lipid profile, and C-reactive protein (CRP). Fibrinogen was measured by the von Clauss method (Instrumentation Laboratory, Bedford, MA, USA).

### 2.4. Plasma PC

The oxidative modification of the plasma proteins was assessed based on carbonyl content, using 2,4-dinitrophenylhydrazine (AppliChem, Darmstadt, Germany), based on the method by Becatti et al. [22]. Briefly, 100 μl of plasma was incubated with 400 μL of 2,4-dinitrophenylhydrazine and precipitated with trichloracetic acid (Sigma–Aldrich, St. Louis, MO, USA). The pellet was washed several times with a 1:1 mixture of ethanol/ethyl acetate (Sigma–Aldrich) and resuspended in 500 μl of guanidine hydrochloride (Sigma–Aldrich). The absorbance was measured at 370 nm using spectrophotometer (Tecan Sunrise, Maennedorf, Switzerland). PC content was expressed as nM/mL of PC per 1 mg of protein. PC levels were measured at four time points: at baseline, at peak exercise, 1 and 24 h after a symptom-limited exercise test.

### 2.5. ELISAs

Human IL-6 and IL-10 (both R&D System, Minneapolis, MN, USA) were measured quantitatively in serum samples at four time points using commercial ELISAs in accordance with the manufacturers' instructions.

### 2.6. CLT

Modified CLT was assayed at four time points using the method described by Lisman et al. [23]. Briefly, diluted citrated plasma was mixed with 15 mM calcium chloride, 0.6 pM human TF (Innovin, Siemens, Marburg, Germany), 12 µM phospholipid vesicles (Avanti Polar Lipids, Alabaster, AL, USA), and 60 ng/mL recombinant tissue plasminogen activator (rtPA; Boerhinger Ingelheim, Ingelheim, Germany). The turbidity was measured at 405 nm at 37°C. CLT was defined as the time from the midpoint of the clear-to-maximum-turbid transition to the midpoint of the maximum-turbid-to-clear transition. Intra-assay and inter-assay coefficients of variation were <8%.

### 2.7. Statistical Analyses

Categorical variables were presented as counts and percentages, while continuous variables were expressed as mean and standard deviation or median with the first and the third quartiles. Categorical variables were analyzed by Pearson's *χ*^2^ or two-tailed Fisher's exact test. Normality was analyzed by the Shapiro–Wilk test. Differences between the AS and control groups were analyzed using the Student's *t*-test or Mann–Whitney *U* test, as appropriate. To compare dependent variables (before and after exercise), the dependent *t*-test was used for normally distributed variables, while for variables with distribution different from normal, the Wilcoxon signed-rank test was used. For analysis of more than two time points (four time points in exercise stress testing), mixed effect models were used, with patient ID as random effect to account for repeated measures. Post hoc comparisons were performed with the Tukey–Kramer HSD test. Associations between variables were calculated using Pearson's or Spearman's correlation coefficients, as appropriate. Linear regression was used to identify predictors of increased PC concentration at peak exercise. A *p*-value <0.05 was considered statistically significant. All statistical analyses were performed using the STATISTICA software (Version 13.3, TIBCO Software, Palo Alto, CA, USA) as well as R version 4.3.1 (R Foundation for Statistical Computing, Vienna, Austria, 2023) with packages “rms” version 6.7-0 and “lme4” version 1.1-34.

## 3. Results

**Figure 1:**
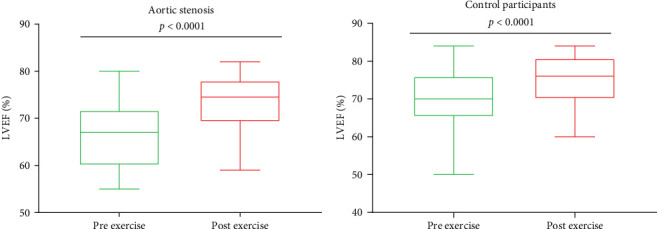
Impact of exercise test on left ventricular ejection fraction in aortic stenosis patients and controls. Box plots showing left ventricular ejection (LVEF) before (green box) and after exercise (red box) (A) in patients with aortic stenosis and (B) in controls. Data presented as median and interquartile range [IQR].

**Figure 2:**
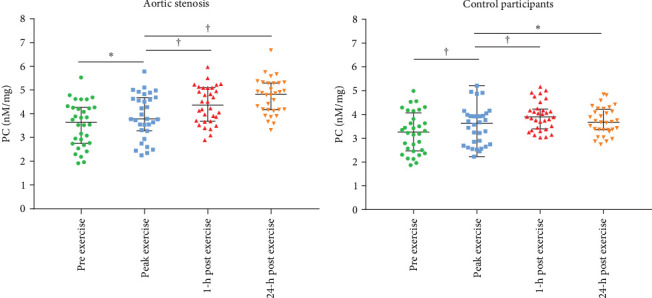
Impact of exercise test on plasma protein carbonyl concentrations in aortic stenosis patients and controls. Dot plots showing plasma protein carbonyls (PC) concentrations before (green dots), during (blue dots) and after exercise (red and orange dots) (A) in patients with aortic stenosis and (B) in controls. Data presented as mean ± standard deviation. Mixed effect models were used, with patient ID as random effect to account for repeated measures. Post hoc comparisons were performed with the Tukey–Kramer HSD test. *⁣*^*∗*^*p*  < 0.05 and ^†^*p*  < 0.001.

Baseline characteristics of AS patients and control individuals are shown in Table 1. AS patients did not differ from controls with regard to demographic or risk factors (all *p*  > 0.05). Patients with AS had slightly higher systolic blood pressure and more frequently used acetylsalicylic acid compared to controls (both *p*  < 0.05; Table 1). AS patients had 7.5-fold higher PG_mean_, 6.7-fold higher PG_max_, and 1.7-fold higher V_max_ compared to controls (all *p*  < 0.0001; Table 1). LVEF increased after exercise stress test compared to baseline values by 13.6% in AS patients and by 8.6% in controls (Figure 1A,B), while transvalvular pressure gradients increased solely in the AS group by 6 mm Hg for PG_mean_ and by 12 mm Hg for PG_max_ (both *p*  < 0.0001). The duration of exercise test was 10% shorter and maximum workload 25% lower in AS patients compared with the control group (Table 1). There were no intergroup differences in routine laboratory parameters (Table 1). Of note, among the recruited AS patients, 13 (40.6%) had moderate AS, while 19 (59.4%) had severe AS. No differences in PC levels were found between AS patients and controls at baseline and peak exercise (both *p* > 0.05). However, AS subjects had 12.8% and 27% higher PC concentrations observed 1 and 24 h after exercise compared to controls (Table 2). In AS patients, PC concentrations were increased during and after exercise, reaching the highest values 24 h post exercise (+30.6%), while in controls 1 h after exercise (+21.9%; Table 2). In AS patients, PC concentration was 8.3% higher at peak exercise compared to baseline (Figure 2A) and was followed by a further increase of PC (+12.8% at 1 h and 20.5% at 24 h; Figure 2A) compared to peak exercise. Likewise, in the control group, PC level was higher at peak exercise (+9.4%) compared to baseline values, as well as 1 and 24 h after exercise (+11.4%, +5.7%, respectively, both *p*  < 0.05) compared to peak exercise (Figure 2B).

**Figure 3:**
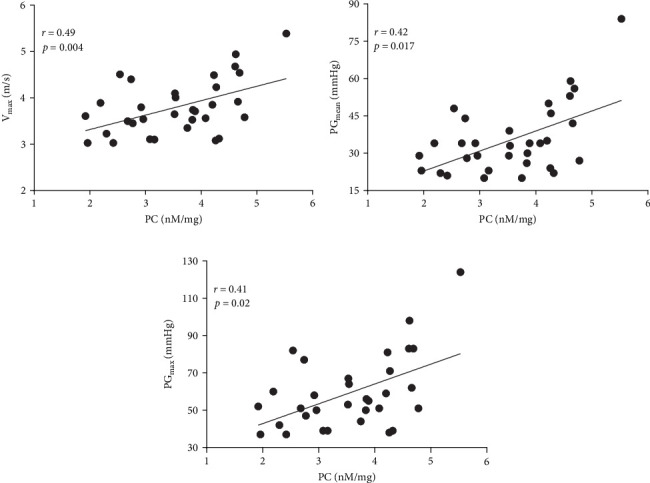
Associations between plasma protein carbonyl concentrations and disease severity in aortic stenosis patients at baseline. Scatterplots represent the correlation between protein carbonyls (PC) concentrations at baseline and (A) peak transvalvular velocity (*V*_max_), (B) mean transvalvular pressure gradient (PG_mean_) and (C) maximal transvalvular pressure gradient (PG_max_). Associations between variables were calculated using Pearson's or Spearman's correlation coefficients, as appropriate. *p*-values of <0.05 were considered statistically significant.

Positive associations between plasma PC concentrations and age were found in both AS and control participants at baseline (*r* = 0.58 and *r* = 82, respectively; both *p*  < 0.005) and remained significant for all time points in both groups (all *p*  < 0.005). In the AS group, PC concentrations solely at baseline correlated with the disease severity measured as *V*_max_ and both transvalvular pressure gradients (PG_mean_ and PG_max_, Figure 3A–C). In the control group, such associations were not observed at baseline, however, 1 h after exercise PC correlated with PG_max_ (*r* = 0.37, *p*=0.038), while PG_mean_ was associated with PC only 24 h post exercise (*r* = 0.38, *p*=0.034).

**Figure 4:**
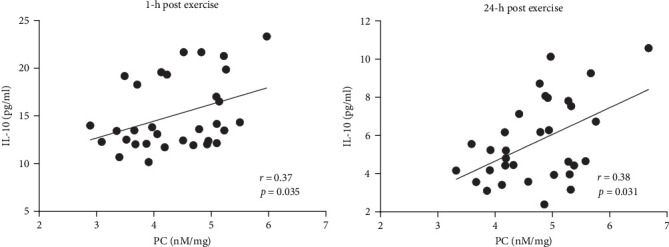
Associations between protein carbonyl and interleukin 10 levels after exercise test in aortic stenosis patients scatterplots represent the correlation between protein carbonyls (PC) concentrations and interleukin 10 (IL-10) levels (A) 1 h post exercise and (B) 24 h post exercise. Associations between variables were calculated using Spearman's correlation coefficient. *p*-values of <0.05 were considered statistically significant.

In AS patients, PC correlated with IL-10 level 1 h and 24 h after exercise (Figure 4A,B), whereas such association in the control group was observed only at baseline (*r* = 0.42, *p*=0.016). No associations between PC levels and IL-6, fibrinogen, CRP, as well as CLT were observed in both groups at any time points.

The univariable linear regression analysis showed that age, PG_mean_, and PC at baseline were associated with PC concentration at peak exercise in both AS and control participants (Table 3).

## 4. Discussion

To our knowledge, this study is the first to show that in asymptomatic patients with moderate-to-severe AS, exercise stress test induced changes in plasma PC levels follow a different pattern as compared to well matched control subjects. Given associations of PC content with increased transvalvular gradients, it might be hypothesized that hemodynamic alterations during exercise test in AS patients enhances oxidative stress and post-translational protein modification detectable in AS is quite fast and maintains even 24 h later, while drops markedly in controls at the same time. Our findings provide evidence that increase transvalvular gradients during exercise could be considered additional factors influencing PC, which could be relevant also in other cardiac disorders. A novel and intriguing observation is a different pattern of PC concentrations observed in AS patients in relation to IL-10.

First, we showed that in patients with AS, plasma PC concentrations at baseline were associated with the disease severity on echocardiography, which was not been reported yet. In control participants, plasma PC concentrations correlated with echocardiographic parameters after exercise stress test, indicating that physical exertion in terms of the duration and intensity of exercise resulted in slightly increased levels of both mean and maximal transvalvular gradients and blood pressure, revealing associations. Especially that both the duration of exercise and maximum workload were significantly greater in control individuals, which may highlight these associations. However, in AS patients such associations were diminished, probably due to physical activity-disturbed hemodynamics. In AS patients, baseline associations between PC levels and echocardiographic parameters were observed, likely reflecting an imbalance between oxidative and antioxidant mechanisms. However, no such correlation was present in healthy individuals, who appear to maintain equilibrium between these processes, suggesting a link between PC dynamics and hemodynamic changes. Specifically, PC levels correlated with PG_max_ one hour post-exercise and with PG_mean_ 24 h post-exercise, indicating a plateau phase possibly attributable to relatively long PC half-life. Moreover, the lack of an increase in IL-10 in response to elevated PC levels may indicate a balance between oxidation and antioxidation mechanisms in healthy individuals. These findings, however, should be interpreted with caution due to the limited sample size and the absence of additional markers, particularly those reflecting antioxidant activity.

In terms of the impact of exercise on oxidative stress, the present study extends the previous findings based on subjects free of AS. It has been demonstrated that exercise induced oxidative stress and increased PC during and after exercise, however, in patients without AS [13–16, 24, 25]. Gochman et al. [13], Bloomer et al. [15], and Wadley et al. [16] showed in young trained subjects that exercise resulted in elevated PC concentrations as a result of intensified training compared to baseline. Similarly, Michailidis et al. [14] and Silva et al. [25] found that exercise bouts in untrained males led to increased protein oxidation compared to pre-exercise values, which corroborate with our findings in AS patients. In our opinion, hemodynamic disturbances following exercise stress test in AS patients could contribute to elevated PC compared to controls, at least in part. However, in our study, we observed higher PC concentrations in both groups compared to other studies [13–15, 24], even if PC assessment was performed using the same method based on the protocol used by Becatti et al. [26]. These discrepancies may be partially explained by younger age of participants, which were about 20 to 30 years old [13–16, 24, 25], various groups of participants including smokers [13], trained [13, 15, 16, 24], and untrained individuals [14, 25], different exercise protocols such as standard maximal exercise test [13], an acute bout of strenuous cardiovascular exercise [14], barbell squat exercise and sprint [15], endurance-based intensified training [16] or eccentric exercise [25], and methodological approach to determine PC concentrations [13–16, 24]. Therefore, further studies, which take these variables into account, will need to be undertaken to enhance the comparability of results.

It has been demonstrated that exercise induces metabolic and immunological responses evidenced by the secretion of inter alia IL-10, which plays a crucial role in both tissue regeneration and energy metabolism, preventing tissue damage [27]. We observed that in AS patients, PC concentrations correlated with the levels of anti-inflammatory cytokine—IL-10 after exercise. These results reflect those of Varamenti et al. [24] who demonstrated gradual increase of IL-10 together with PC concentration during and after exercise in female athletes. This observation may suggest that in patients with AS, transvalvular gradients lead to the secretion of IL-10 providing protection against tissue damage. Furthermore, in contrast to healthy individuals, an increase in IL-10 correlating with higher PC may reflect a subclinical inflammatory process characteristic of AS patients.

We found no associations between plasma PC concentrations and IL-6 levels in both groups. However, serum IL-6 levels in patients with severe AS were low, similar to those observed in apparently healthy volunteers [28], which can result in the lack of associations between PC and IL-6. Moreover, previous reports on the association between PC concentrations and circulating levels of the proinflammatory cytokine—IL-6 are inconsistent [29–31]. Dayhoff-Brannigan et al. [29] showed a positive correlation between PC and IL-6 levels in a cohort study of 739 women aged ≥65 years, while no such association was noted by Song et al. [30] and Szelényi et al. [31] in hemodialysis patients and in subjects with hypertension, respectively.

Both inflammation and hemostatic alterations have been documented to be involved in AS progression [32–35]. Our previous studies showed that expression of TF, FXIII, and fibrin, as well as PAI-1 was accompanied by macrophages infiltration mainly in the area of fatty-calcium deposits [32–35]. We also observed that the proinflammatory stimulation of valvular interstitial cells (VICs) with TNF-*α* leads to upregulation of TF, thrombin, FVII and FX/FXa expression on both protein and mRNA levels [36], as well as increased PAI-1 concentrations in VICs supernatants [37]. On the other hand, IL-10 as an anti-inflammatory cytokine counteracts the effects of proinflammatory cytokines like IL-6 and TNF-*α* [38], reduces oxidative stress, and may limit the inflammation-induced thrombosis by enhancing fibrin breakdown and downregulation of TF expression [39–41]. In patients with AS, this balance is disturbed, and antioxidant mechanisms are impaired due to the progression of the disease. Therefore, in this group, IL-10 may serve as an additional anti-inflammatory response to exercise stress, while in healthy individuals the balance between oxidative and anti-oxidative mechanisms is sufficient to neutralize exercise stress effects.

In contrast to the study by Siudut et al. [7] in which in patients with symptomatic severe AS scheduled for valve replacement, PC concentrations correlated with prolonged CLT as well as the disease severity, we did not observe such associations. This discrepancy may be explained by the fact that Siudut et al. [7] investigated patients with more advanced AS associated with a more pronounced prothrombotic and hypofibrinolytic state, while in our study patients were asymptomatic with moderate-to-severe AS and only 59.4% of patients in our cohort represented severe form of AS. It might be speculated that unfavorable fibrin clot structure related to increased PC, particularly of fibrinogen, impairs clot lysis [42]. Fibrinogen is 20 times more susceptible to oxidative modification than albumin, particularly to carbonylation [42]. Since PC is nonreversible and leads to protein dysfunction, fibrinogen carbonylation results in the formation of denser clots with thinner fibers, increased cross-linking, and decreased lysability [42]. However, coagulation factors and fibrinolysis inhibitors, such as PAI-1 and thrombin activatable fibrinolysis inhibitor (TAFI), have been shown to undergo carbonylation [26, 43]. Carbonylation of PAI-1 and TAFI has been shown to influence their activity and stability, potentially impacting fibrinolysis [43]. Interestingly, a recent study by Mróz et al. [44] demonstrated in patients with stable coronary artery disease that enhanced PC was associated with increased levels of PAI-1 and TAFI, as well as with reduced plasma fibrin clot permeability. Further research is needed to clarify the mechanisms connecting increased PC and hypofibrinolysis in AS. However, in our opinion, carbonylation of fibrinogen was insufficient to be associated with CLT, which may result from too short exercise stress test duration.

Notably, antioxidant agents and various diets can impact oxidative stress levels and, consequently, the degree of PC. A study by da Silva et al. [45] demonstrated that taurine supplementation reduced PC levels in young male individuals following eccentric exercise compared to the placebo group. Similarly, a study has shown that a Mediterranean diet supplemented with coenzyme Q10 reduces postprandial oxidative stress, as indicated by decreased PC levels, compared to a Mediterranean diet alone and a diet rich in saturated fatty acids in elderly men and women [46]. In a randomized clinical trial, Pivovarova-Ramich et al. [47] demonstrated that both animal-based and plant-based protein diets similarly reduced PC levels after a 6 weeks of intervention in individuals with type 2 diabetes. This reduction was accompanied by significant improvements in glycemic control, as well as reductions in blood lipids, blood pressure, and inflammatory cytokines [47]. In contrast, Vileigas et al. [48] found in animal model that obese rats fed a high-fat Western diet had significantly elevated PC concentrations compared to control rats.

## 5. Study Limitations

This study has several limitations. First, the number of study participants was limited, which may have prevented some associations from being revealed. However, this is a hypothesis-generating report and represents typical asymptomatic patients with moderate-to-severe AS, who were able to perform exercise testing. Second, our results cannot be likely extrapolated to individuals with mild AS, and further studies are needed to assess potential differences in PC changes induced by exercise in such individuals. Additionally, all participants were Caucasian, so the results may not be applicable to other populations. Third, in our study, plasma PC levels in AS patients did not decrease 24 h post exercise, therefore, additional PC measurements should be performed to determine when PC concentrations return to baseline values in asymptomatic AS. Furthermore, due to a limited number of patients, severe multicollinearity among many variables, and the results of the univariable linear regression model, multivariable analysis was not performed. It would also be of interest to evaluate antioxidant capacity to examine the potential crosstalk between antioxidant status and oxidative stress during exercise testing in asymptomatic AS patients. Additionally, data on antioxidant intake and different types of diets were not collected during participant recruitment, since these factors were beyond the scope of the current study. Finally, in this study, exercise testing was performed on a bicycle ergometer, thus studies using another exercise protocols might lead to different observations, though given associations of PC with gradients it appears unlikely.

## 6. Conclusions

Our study suggests that in asymptomatic patients with moderate-to-severe AS, hemodynamic changes during exercise stress test lead to increased oxidative stress, resulting in PC, which is associated with IL-10 release in the post-exercise period. Enhanced PC following exercise is also prolonged suggesting dysregulation in antioxidant mechanisms in this disease without coexistent evident atherosclerotic vascular disease. Further studies are needed to elucidate molecular mechanisms behind this interesting observation, which suggests a previously unknown factor affecting PC in humans.

## Figures and Tables

**Table 1:** Baseline characteristics of the study groups.

Variable	AS patients (*n* = 32)	Controls (*n* = 32)	*p*-Value
Age, years	63.3 ± 13	63.3 ± 11	0.99
Male, *n* (%)	15 (46.9)	17 (53.1)	0.61
BMI (kg m^−2^)	29.5 ± 4.2	28.2 ± 4.6	0.23
Risk factors			
Hypercholesterolemia, *n* (%)	23 (71.9)	20 (62.5)	0.42
Diabetes mellitus, *n* (%)	7 (21.9)	4 (12.5)	0.51
Current smoking, *n* (%)	2 (6.3)	2 (6.3)	0.69
Intima-media thickness (mm)	0.74 ± 0.19	0.71 ± 0.15	0.41
Medications, *n* (%)			
Beta-blockers, *n* (%)	19 (59.4)	12 (37.5)	0.08
Acetylsalicylic acid, *n* (%)	18 (56.3)	10 (31.3)	0.044
ACE inhibitors, *n* (%)	12 (37.5)	15 (46.9)	0.45
Statins, *n* (%)	23 (71.9)	20 (62.5)	0.42
Blood pressure (mmHg)			
Systolic pressure	143 ± 16	135 ± 12.5	0.034
Diastolic pressure	74 [70–83]	80 [70–82]	0.7
Echocardiographic parameters			
PG_mean_ (mmHg)	34 [25–43]	4 [3–6]	<0.0001
PG_max_ (mmHg)	54 [46–69]	7 [5.0–10]	<0.0001
*V*_max_ (m/s)	3.80 ± 0.59	1.41 ± 0.29	<0.0001
LVEF pre-exercise (%)	66 ± 7	70 ± 8	0.68
LVEF post-exercise (%)	75 [70–78]	76 [70–81]	0.27
Exercise data			
Maximal workload (W)	75 [75–86]	100 [75–125]	0.0017
Duration (min)	9 [7.5–10.3]	10 [8.5–13.4]	0.0085
Laboratory investigations			
Fibrinogen (g/L)	3.3 [2.6–3.8]	3.0 [2.7–3.5]	0.35
Creatinine (mmol/L)	73.8 ± 12.4	77.3 ± 14	0.28
C-reactive protein, mg/L	1.2 [0.7–2.8]	1.6 [0.9–3.0]	0.61
Glucose (mmol/L)	5.4 [5.2–6.2]	5.4 [5.0–5.8]	0.16
Total cholesterol (mmol/L)	5.0 [4.4–5.7]	5.0 [4.3–6.5]	0.6
LDL-cholesterol (mmol/L)	3.0 [2.4–3.7]	3.1 [2.4–4.2]	0.42
HDL-cholesterol (mmol/L)	1.5 ± 0.4	1.5 ± 0.4	0.15
Triglycerides (mmol/L)	1.2 [0.9–1.7]	1.2 [0.9–1.5]	0.5

*Note:* Data presented as numbers (percentages), mean ± standard deviation or medians [interquartile range]. Differences between the AS and control groups were analyzed using the Student's *t*-test or Mann–Whitney *U* test, as appropriate. *p*-values of <0.05 were considered statistically significant.

Abbreviations: ACE inhibitors, angiotensin-converting enzyme inhibitors; AS, aortic stenosis; LVEF, left ventricular ejection fraction; PG_max_, maximal transvalvular pressure gradient; PG_mean_, mean transvalvular pressure gradient; V_max_, peak transvalvular velocity.

**Table 2:** Plasma protein carbonyls concentrations in patients with aortic stenosis and in controls during exercise test.

Variable	Time point	AS patients (*n* = 32)	Controls (*n* = 32)	*p*-Value
PC (nM/mg)	Pre exercise	3.6 ± 0.94	3.2 ± 0.86	0.17
	Peak exercise	3.9 ± 0.94	3.5 ± 0.81	0.09
	1 h post exercise	4.4 ± 0.78	3.9 ± 0.61	0.016
	24 h post exercise	4.7 ± 0.74	3.7 ± 0.58	<0.0001

*Note:* Data presented as mean ± standard deviation. Differences between the AS and control groups were analyzed using the Student's *t*-test. *p*-values of <0.05 were considered statistically significant.

Abbreviation: PC, protein carbonyls.

**Table 3:** Determinants of plasma protein carbonyls concentration at peak exercise in all participants.

Univariable			
Variable	Estimate	SE	*p*-value
Age, per 10 years	0.398	0.050	<0.0001
PG_mean_, per 10 mmHg	0.685	0.288	0.021
PC at baseline, per 1 nM mg	0.227	0.104	0.008

*Note: N* = 64, *p*-values of <0.05 were considered statistically significant.

Abbreviations: PC, protein carbonyls; PG_mean_, mean transvalvular pressure gradient; SE, standard error.

## Data Availability

The data that support the findings of this study are available from the corresponding author upon reasonable request.
